# Interlinkage between health workforce availability and socioeconomic status in rural and remote Australia

**DOI:** 10.1371/journal.pone.0321198

**Published:** 2025-04-11

**Authors:** Ellen McDonald, Ross Bailie, Peter Radchenko, K. Shuvo Bakar

**Affiliations:** 1 School of Public Health, Faculty of Medicine and Health, The University of Sydney. Camperdown, New South Wales,; 2 Sydney Business School, The University of Sydney, Darlington, New South Wales; The World Bank Group, Canada

## Abstract

**Introduction:**

Australians living in rural and remote areas experience a higher burden of disease compared to their urban counterparts, whilst having poorer access to essential health services. Socioeconomic status and health workforce shortages are important influences on health status and access to care in these areas. This research aims to provide a local-level analysis of the association between local government area (LGA) indicators of socio-economic status and health workforce availability to enhance understanding of rural and remote workforce distribution patterns.

**Methods:**

Data were extracted from the Australian Bureau of Statistics and the Department of Health and Aged care, which encompassed demographic factors, socioeconomic indicators and counts for medical practitioners, allied health workers and nurses and midwives within non-metropolitan local government areas. Generalised Additive Models with Generalised Estimating Equations (GEE-GAMs) were used to test for an association between socioeconomic status (SES) and the World Health Organisation’s definition of health workforce deficit.

**Results:**

The odds of being in deficit of nurses and midwives increased with increasing SES. No significant association between SES and medical practitioners or allied healthcare workers was found. Very remote areas were less likely to have a deficit of allied health professionals than inner regional areas, and the same was true for nurses and midwives in both remote and very remote areas.

**Conclusions:**

The findings suggest that health workforce policies that target areas of need based on SES, may have contributed to better availability of nurses and midwives in these locations, but not significantly so for medical practitioners or allied health professionals. Further research is required to investigate the relative success of workforce policies in addressing health need in relation to SES and remoteness.

## 1. Introduction

Globally, those living in rural and remote areas tend to suffer from poorer health outcomes when compared to their urban counterparts and have relatively unfavourable social determinants of health [1]. Australia is no exception to this phenomenon, with rural and remote areas having higher rates of hospitalisation, death and injury when compared to major cities, and lower rates of education and employment [2]. National statistics show that burden of disease from conditions such as coronary heart disease, type 2 diabetes and lung conditions increases with location remoteness [2], and subsequently rural and remote populations have been identified as a priority in Australia’s National Preventative Health Strategy [3]. In this paper, we refer to rural and remote areas as those outside of major cities, as defined by the Australian Bureau of Statistics (ABS).

Despite the national prioritisation of rural and remote health, access to healthcare in these areas continue to be limited [4]. Consistent with global trends [1], those living in rural and remote areas of Australia have poorer access to health services compared to those living in major cities [2]. While rural and remote areas face unique socio-economic and geographical challenges that contribute to health service limitations, a deficit of trained health professionals is central to the inadequate availability of health services in these areas [1]. In Australia, the number of full-time equivalent (FTE) healthcare workers per head decreases with increasing location remoteness [4], demonstrating that the health workforce is being inversely distributed in relation to relative need [5]. While various programs and incentives exist to attract healthcare workers to rural and remote areas in Australia [6], evidence of effectiveness is limited [7,8], and the health workforce remains unevenly distributed. Research into the determinants of health workforce availability in rural and remote Australia is needed to guide evidence-based policy and address the imbalanced distribution of the health workforce.

The current literature base surrounding the determinants of health workforce availability in rural and remote Australia mainly describes factors that influence retention and recruitment.

Current studies suggest that personal factors such as a rural background or extended training in rural areas increases the likelihood of healthcare professionals working rurally [8,9,10,11,12]. The Rural Health Multidisciplinary Training program in Australia facilitates rural training and recruitment of rural students, and has been shown to be an important contributor to addressing rural and remote health workforce shortages [13].

Other determinants of rural and remote health workforce retention and recruitment in Australia include place-based and workplace factors. Ineffective management, poor work environments and lack of career progression in rural and remote settings is detrimental to healthcare worker retention [7,12,14,15]. Place based factors such as increased remoteness, community deprivation, lack of facilities and social isolation are also detrimental to rural and remote health workforce retention [11,14,15]. Determinants associated with increased retention include community engagement and having access to family support, childcare and appropriate housing [7,14,16,17]. However, the link between these determinants and effective intervention is not strong [7,8], and primary sources of evidence are mostly restricted to local settings, which limits the generalisability of this evidence.

In 2023, Yisma et al. analysed the distribution of occupational therapists, physiotherapists and podiatrists across Australia by socioeconomic status (SES) of areas [18]. SES was represented by index of relative socioeconomic advantage and disadvantage (IRSAD), and the density of occupational therapists, physiotherapists and podiatrists was found to decrease with decreasing IRSAD quintile (which equates to decreasing SES) [18]. No other literature within Australia could be identified that investigates the relationship between healthcare worker availability and local area-level SES, with studies that consider local area-level SES focusing on health service utilisation [19,20], rather than health workforce availability.

Turning to the international context, studies in England found a “pro-rich” relationship between general practitioner density and socioeconomic status after adjusting for need [21,22,23]. In one study, this relationship also held true for paramedics, while the opposite was true for nursing staff [21]. Looking even more broadly, Cookson et al. found that health workforce availability is inversely associated with area socioeconomic disadvantage in most low and middle-income countries (i.e., health workforce availability decreases with increasing socioeconomic disadvantage), however in high income countries the same is true only after adjustment for need [24].

Important geographical and socio-economic differences exist between Australia and other parts of the world, particularly in non-metropolitan settings, meaning the transferability of results from international studies to Australia may be limited. The findings from Yisma et al. are limited in transferability and generalisability due to inclusion of only three allied health professions, and the analysis not being specific to the rural and remote setting [18]. While the current evidence base suggests there is an association between the availability of specific healthcare workers and the socioeconomic status of areas [18,21–23], this association needs to be examined in the rural and remote Australian setting, across a broader range of health professions.

Investigating the relationship between local area-level SES and health workforce availability should improve understanding of the distribution of Australia’s rural and remote health workforce, and provide insight into the extent to which health workforce policies have been effective in improving availability of different types of health professionals in areas higher need as reflected by SES and rurality. Those living in areas of lower SES in the rural and remote setting may face intersecting disadvantage and will likely have higher demands for health services, given the well-known association between low SES and poor health [25,26], and increased rurality and poor health [2]. Knowledge relating to the current relationship between local area-level SES and rural and remote health workforce availability may inform policy and health workforce planning that aims to distribute healthcare professionals to areas of highest need.

This paper explores the association between local area-level SES and a 2016 WHO definition of health workforce deficit (see details in the methods section below) in areas outside of major cities, per the Australian statistical geography standard remoteness structure. The influence of area remoteness is explored and adjusted for in the statistical analysis. We focus on three categories of health workers: medical practitioners, allied health workers and nurses and midwives, as these professionals make up an essential portion of the health workforce [1,27]. For this paper, areas outside of major cities including inner regional, outer regional, remote and very remote areas (per the Australian statistical geography standard remoteness structure) will be referred to as rural and remote areas.

## 2. Methods

### 2.1 Data

The Australian Bureau of statistics (ABS) census data was used to extract socio-economic indicators for areas (SEIFA) from 2013 to 2021, at local government area (LGA) level across rural and remote Australia [28]. SEIFA scores rank areas by socio-economic advantage and disadvantage through variables measured in census data [29]. Age distributions for rural and remote LGAs were also sourced from the ABS, using data-explorer [30]. The proportion of residents aged over 85 years old and aged under 5 years old in each LGA were calculated. The following data were collected from the Health Workforce Data Tool [31], provided by the Australian Government Department of Health and Aged Care for the same time periods (2013–2021) and by LGA:

- Population size.
- Number of all full time equivalent (FTE) medical practitioners that are registered with the Australian Health Practitioner Regulation Agency (AHPRA) and working.
- Number of all FTE nurses and midwives (as a singular total) working and registered with AHPRA.
- Number of FTE allied health professionals registered with AHPRA and working. As is consistent with the Australian Institute of Health and Welfare [4], this includes Aboriginal and Torres Strait Islander health practitioners, chiropractors, Chinese medicine practitioners, medical radiation practitioners, occupational therapists, optometrists, osteopaths, pharmacists, physiotherapists, podiatrists, paramedics and psychologists.
- Remoteness area, measured by the Australian statistical geography standard remoteness structure.

### 2.2 Study variables

The outcome variable is binary, with each LGA being coded as either being in deficit or not being in deficit of the categories of health care worker of interest, i.e., “medical practitioners”, “nurses and midwives”, and “allied health professionals”. Thresholds to define a “deficit” were extracted from WHO’s 2016 report on health workforce requirements. This report concluded that 4.45 medical practitioners, and 4.45 nurses and midwives are required per 1,000 people to achieve health related sustainable development goals [32]. Therefore, under 4.45 FTE workers per 1,000 people was defined as a deficit for medical practitioners and nurses and midwives.

The WHO health workforce requirements report recognised the importance of allied health professionals but did not define a minimum workforce requirement for this profession due to difficulty centralising data [32]. Due to a lack of other literature surrounding the required quantity of allied health professionals, the 2020 Australian national average of allied health professionals per 1,000 population was used to define a deficit in this category, which is 5.94 FTE allied health professionals per 1,000 people [4].

Index of relative socioeconomic advantage and disadvantage (IRSAD) was the SEIFA score chosen to represent SES in this analysis, as it considers elements of both advantage and disadvantage, and encompasses the broadest range of variables [29]. IRSAD will be measured in quintiles, as is consistent with national [18,33], and international literature [22,23] examining the socioeconomic status of areas in a similar context. A higher IRSAD quintile represents higher SES.

The following variables were included as covariates:

- ◦Remoteness area (RA), measured by the Australian statistical geography standard remoteness structure.
- ◦Age distribution variables:
  - ▪proportion aged under 5 per 1,000 population.
  - ▪proportion aged over 85 per 1,000 population.
- ◦Time in years from 2013 to 2021.

**Fig 1:**
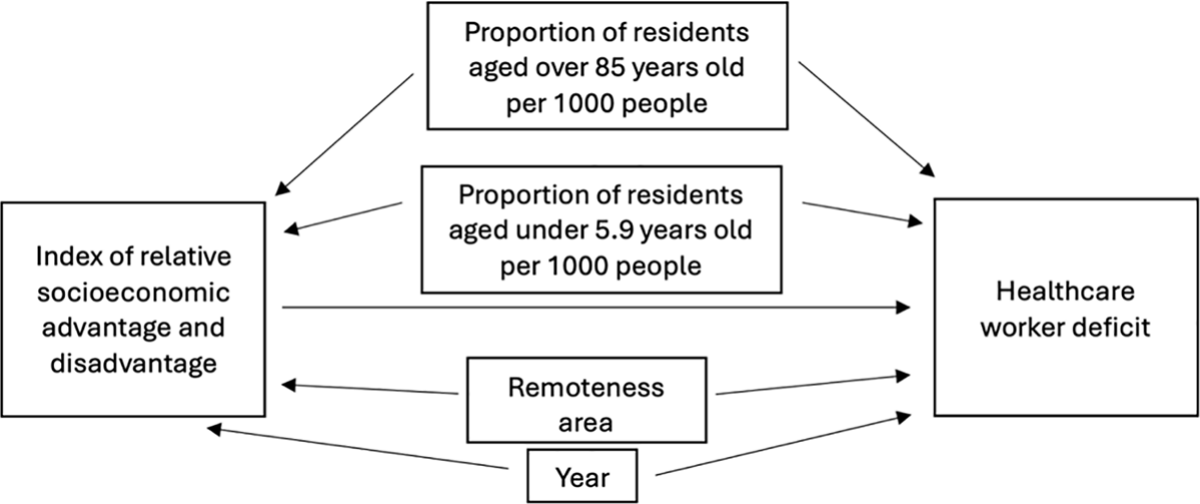
Diagram depicting possible relationships between the included covariables and the outcome variable (health workforce deficit), and the exposure variable (index of relative socioeconomic advantage and disadvantage quintile).

These covariates were included based on the assumptions illustrated in Fig 1, which were guided by current literature. National statistics demonstrate that the remoteness of an area is independently associated with both SES [1,2] and health workforce availability [4], making RA an important covariate to adjust for to avoid possible confounding. Year was also included as a covariate, as time may influence both health workforce availability and IRSAD scores through events such as the covid-19 pandemic. Age distributions from two extreme tails of the LGA population, i.e., less than 5 and over 85 years old were also included as covariates, due to possible relationships between age and local area-level SES [26], which may influence health workforce availability through workforce retention factors such as availability of childcare services [16] and community engagement [14].

### 3.3 Statistical methods

Data were examined using summary statistics, and repeated observations were deleted. Examination of diagnostic plots showed a non-linear relationship between the continuous variables and the binary outcome variable, which prompted the development of a non-linear additive structure in our model for the continuous variables. More specifically, we used Generalised Additive Models (GAMs), which allow for inclusion of non-linear continuous variables by use of cubic regression splines [34]. GAMs were created for each healthcare profession.

Due to the clustering effects in the data, generalised estimating equation (GEE) estimation was also implemented. The data were considered to be clustered on the basis that separate observations from a single LGA are likely to be more correlated than observations from different LGAs [35]. This clustering violates the independence assumption of GAMs, making GEEs a necessary addition to address this violation [36]. This decision was supported by the large intra-cluster correlation values within each GEE model. GEEs address clustering by assuming a correlation structure within the dataset, which allows for calculation of robust standard errors [36]. GEEs are robust to misspecification of correlation structures [37], and were selected as the most appropriate method to account for clustering as they allow for population averaged estimations.

To create the GEE-GAM models, the splines of the continuous variables were extracted from the GAMs and applied to GEE-GLMs. Similar techniques of developing GEE-GAMs have been shown to be successful in literature [38]. The QIC (quasi-likelihood under the independence model criterion) was used to identify the best and final GEE-GAM for all workforce categories. Age distribution variables were considered for removal from the final GEE-GAM models if they did not cause an increase in the QIC value or a significant change in the IRSAD quintile coefficients after removal. RA and year variables were not considered for removal due to their strong contextual relevance to the model.

The model selection process was completed with both exchangeable and autoregressive correlation structures for each GEE-GAM, and the correlation structure for each final model was selected based on the lowest QIC value. Effect modification between year and age distribution variables was also tested in the final model and considered for inclusion if interaction terms were significant. QIC values were compared between the initial GEE-GLMs and the final GEE-GAMs to ensure the best model was selected.

We used R programming language to implement the models [36,39], and used “glm”, “mgcv” [34,40] and “geepack” [41] packages to get model-based results. Table B and C in S1 File includes model-based output for crude and adjusted GLMs for the health professional categories considered in this paper.

## 4. Results

### 4.1 Descriptive statistics

A total of 3,294 observations were collected from 368 rural and remote LGAs across Australia, over nine years. There were no missing data. IRSAD quintile 5 (most advantages quintile) contained the smallest proportion (8.4%) of observations, while quintiles one to four each contained between 20% to 24% of the remaining observations. The highest proportion of observations were from inner regional Australia (41.6%), and the lowest proportion were from remote Australia (11.3%).

There was an overall 15.5% relative decrease in the proportion of LGA’s in deficit of allied health professionals between 2013 and 2021 (Table 1). The proportion of LGAs in deficit of medical practitioners in 2013 had decreased by 3% in 2021, which is the smallest relative change amongst all health profession categories. Nurses and midwives had a 9.6% relative decrease in the proportion of LGAs in deficit between 2013 and 2021.

**Table 1:** The relative change in proportion of deficit for three categories of healthcare professions, between the years 2013 and 2021 across 368 rural and remote Australian local government areas, by remoteness area and index of relative socioeconomic advantage and disadvantage (IRSAD).

Relative change in proportion of deficit between 2013 and 2021 (%)			
	Allied Health Professionals	Medical Practitioners	Nurses and Midwives
Remoteness Area			
Inner regional Outer regional Remote Very remote	-13.7% -7.8% -15.1% -37.0%	-6.1% -1.0% 3.4% -7.9%	-2.0% -0.8% 0.0% 0.0%
IRSAD			
Quintile 1 Quintile 2 Quintile 3 Quintile 4 Quintile 5	-15.5% -14.9% -13.5% -16.5% -15.9%	-2.7% -3.6% -8.7% 2.3% 1.8%	0.0% -10.1% -44.5% -3.3% -1.2%
Total relative change	-15.5%	-3.02	- 9.6%

Relative change = (Proportion of deficit in 2021 - Proportion of deficit in 2013)/ Proportion of deficit in 2013

### 4.2 Model-based results

**Fig 2:**
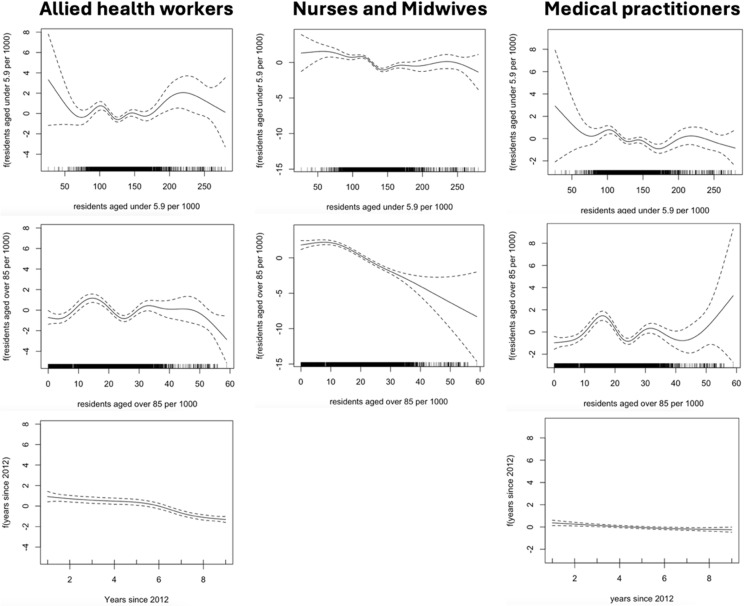
Smooth functions for continuous covariates included in the GEE-GAM (generalised estimating equations applied to a generalised additive model).

All GEE-GAMs had lower QIC values than their corresponding GEE-GLMs, and were therefore selected as final models. The final GEE-GAM for allied health professionals included all the covariates, as listed in Table 2. All continuous covariates were observed to have a non-linear relationship with the logit of the outcome variable, and were therefore included as smoothed variables, which are plotted in Fig 2. Age distribution variables remained in the final model as their exclusion increased the QIC score. There was no evidence of effect modification between year and age distribution variables. The C-statistic for this model is 0.77, which is greater than the null model (0.5). The QIC value for this model (1652) is lower than that of the null model (1824). Table A in S1 File provides the model-based results.

**Table 2:** Results from the final Generalised Additive Model with Generalised Estimating Equations for each healthcare profession.

	Allied health professionals	Nurses and midwives	Medical practitioners
IRSAD^#^	OR(SE)^##^	OR(SE)^##^	OR(SE)^##^
Quintile 2 Quintile 3 Quintile 4 Quintile 5	1.01(0.39) 0.96(0.42) 0.70(0.47) 0.65(0.56)	1.34(0.16). 1.43(0.18). 1.65(0.20)^*^ 2.54(0.29)^**^	0.94(0.30) 1.12(0.34) 0.81(0.35) 0.93(0.41)
Area remoteness			
Outer regional Remote Very remote	1.39(0.42) 0.65(0.47) 0.30(0.46)^**^	0.64(0.32) 0.12(0.61)^**^ 0.28(0.41)^**^	3.04(0.47) 1.56(0.50) 1.18(0.44)
Year	–	1.01(0.01)	–
Intercept OR(SE)	26.50(0.51)	0.15(0.25)	8.74(0.35)
	**edf** ^**###**^	**edf** ^**###**^	**edf** ^**###**^
f(Year)^####^	4.33^***^	–	1.58^**^
f(Proportion aged over 85/ 1000)^####^	7.72^***^	3.91^***^	8.01^***^
f(Proportion aged under 5/ 1000)^####^	8.19^***^	8.23^***^	8.12^***^
Performance statistics			
C statistic	0.77	0.76	0.73
QIC	1652	2331	2016

Significance key: (p<0.001 = ^***^) (p<0.01 = ^**^) (p<0.05 = ^*^) (p<0.1 =.) (p≥0.1 = no symbol)

# IRSAD = Index of relative socioeconomic advantage and disadvantage

## OR(SE) = Odds ratio(standard error)

### edf = estimated degrees of freedom

#### f(x) = functional form of x, and x implies as a variable or predictor in the model.

The estimated odds ratios calculated for each IRSAD quintile (Table 2) have large robust standard errors, and there is no statistical evidence (p>0.1) of association between IRSAD quintile and allied health professional deficit. However, there is strong evidence (p<0.01) that LGAs in very remote areas have on average 70% lower odds of being in deficit of allied health professionals compared to LGAs in inner regional areas, after adjusting for the covariates listed in Table 2.

The GEE-GAM model for medical practitioners included all covariables, and all continuous variables were included as smoothed functions (Fig 2) due to non-linearity. The age distribution variables increased the QIC value when removed from the model and were therefore included. There was no evidence of effect modification between the age distribution variables and year. The C-statistic for this model was 0.73 which is higher than that of the null model (0.5). The QIC for the final model (2016) was also lower than that of the null model (2140). The model showed no statistical evidence of association between IRSAD quintile and medical practitioner deficit, due to large robust standard errors. There was also no statistical association between area remoteness and medical practitioner deficit.

The GEE-GAM model for nurses and midwives also included all variables, with both age distribution variables being included as smoothed terms (Fig 2). Year was included as a linear term in the model after graphical inspection. The age distribution variables were not removed from the model, as their presence lowered the model’s QIC value. There was no evidence of effect modification between either of the age distribution variables and year.

This model showed a significant association between nurse and midwife deficit and IRSAD quintile. Strong statistical evidence (p<0.01) demonstrated that on average, LGAs in quintile 5 had 2.54 times the odds being in deficit of nurses and midwives compared to LGAs in quintile 1. There was also evidence (p<0.05) to show that LGAs in IRSAD quintile 4 had on average 1.65 times the odds of being in deficit of nurses and midwives compared to LGAs in IRSAD quintile 1. Additionally, there is weak evidence (p<0.1) to show that on average LGAs in IRSAD quintile 2 and 3 had respectively 1.34 and 1.43 times the odds of being in deficit of nurses and midwives when compared to LGAs in IRSAD quintile 1. All conclusions were after adjustment for the covariates listed in Table 2, and after accounting for clustering.

Additionally, strong evidence shows that LGAs in remote areas have on average 88% lower odds of being in deficit of nurses and midwives than LGAs in inner regional areas, and that LGAs in very remote areas have 72% lower odds of being in deficit of nurses and midwives than LGAs in inner regional areas, after adjusting for the included covariates.

## 5. Discussion

Our findings illustrate a large overall deficit of both medical practitioners and allied health professionals in rural and remote areas, which is consistent with national statistics [4]. Conversely, a much smaller deficit of nurses and midwives exists, suggesting that nurses and midwives are more adequately distributed within rural and remote areas than medical practitioners and allied health professionals. All three categories of health professionals have an overall relative decrease in deficit of health professionals between 2013 and 2021, however the magnitude of the relative reduction in deficit varies between health professions. Medical practitioners have a very small relative decrease in deficit over time, while allied health professionals and nurses and midwives had a much larger relative decrease in deficit. This may indicate that health workforce distribution policies have been more effective for allied health professionals and nurses and midwives than for medical practitioners over the period 2013–2021.

Our findings indicate that in the rural and remote Australian setting, the availability of both medical practitioners and allied health workers is not associated with local area-level SES. This is inconsistent with findings from international and national sources, which show that decreased local area-level SES is associated with decreasing density of general practitioners [21,23] and various allied health professionals [18,21]. Unlike these sources, our findings are specific to the rural and remote setting, which may suggest that the influence of local area-level SES on retention and recruitment for these professions differs between non-metropolitan and metropolitan areas. The lack of association found in this paper may indicate that in the rural and remote setting, medical practitioners and allied health workers are not deterred by socioeconomic factors relating to their area of practice, or that existing health workforce policies are to some extent addressing the higher level of need in areas of lower SES. Alternatively, the widespread deficits among these health professions across rural and remote Australia could be overshadowing the association between SES and deficit, and perhaps an association would emerge if rural and remote areas had lower overall levels of deficit.

The difference between our findings and that of existing literature may also be due to differences in analysis. Nussbaum et al. [21], Fisher et al. [23], and Yisma et al. [18] examined density of healthcare professionals rather than deficit, and their findings were specific to individual professions. The grouping of professions in this analysis may have masked profession specific associations, with the lack of association found possibly being indicative of heterogeneity within different specific allied health and medical professions. Nussbaum et al., and Fisher et al. also adjusted for need in their analyses, with Fisher et al. finding no association between general practitioner density and area disadvantage prior to adjusting for need. This may also explain differences in results.

Our findings relating to the association between SES and nurse and midwife deficit differs from that found among medical practitioners and allied health workers. Our results show that SES is associated with nurse and midwife deficit, and that areas of lower SES are less likely to be in deficit of nurses and midwives when compared to areas of higher SES. These results are supported by the literature in England, which found an increased proportion of nurses per 10,000 patients in areas of higher disadvantage [21]. Our results also show that remote and very remote areas were significantly less likely to have a deficit of nurses and midwives compared to inner regional areas [21]. This phenomenon was mirrored among allied health professionals, with very remote areas being less likely to be in deficit of allied health workers compared to inner regional areas.

Lower SES and increased remoteness being associated with lower odds of nurse/midwife deficit suggests that nurses and midwives are present in areas of relatively higher need, and that current health workforce policies may have been more effective for these professions. However, this is concurrent with a widespread deficit of medical practitioners and allied health professionals in rural and remote areas. An adequate supply of nurses and midwives in the absence of medical practitioners and allied health professionals is not necessarily indicative of adequate health service availability [42]. These results may be illustrating an increased reliance on nurses and midwives in rural and remote areas and areas of lower SES (due to a shortage of medical practitioners and allied health professionals), with nurses and midwives filling a wider range of health professional roles in these locations [23,32].

### 5.1 Limitations

The use of IRSAD as a proxy for SES is a limitation of the models created. While IRSAD is a widely used measure of SES that undergoes validation by the ABS [28], socioeconomic status remains a difficult concept to quantify. IRSAD ranks relative socioeconomic advantage and disadvantage, meaning LGAs in lower IRSAD quintiles are not necessarily of low socioeconomic status, but are of lower SES compared to LGAs in higher quintiles [29]. Hence, the conclusions of this study cannot be equated to actual SES, but relative SES.

Another key limitation of the study is the lack of detailed data on the breakdown of health workforce professions. For example, nursing and midwifery data are combined by AHPRA, despite their distinct scopes of practice. These professions often have separate roles and are frequently the first point of contact for providing essential services in health and primary care, health promotion, and disease prevention. Therefore, analysing these two professions separately would provide a more meaningful perspective for policy implications.

Analysing health workforce deficit as a binary outcome rather than as a continuous measure also introduces model limitations. The use of a binary outcome variable means that the magnitude to which an observation is above or below the deficit threshold is not accounted for, and more detailed associations cannot be uncovered. The efficacy of the final models is also dependent on the accuracy of the deficit thresholds defined. The lack of evidence surrounding the minimum requirements for allied health professionals means deficit in this category was based on the national average of FTE allied health workers in 2020, which may be an inaccurate representation of deficit. The minimum health workforce requirements estimated by the World Health Organisation are not specific to the rural and remote Australian setting, and therefore may decrease accuracy of the thresholds defined for medical practitioners and nurses and midwives.

Additionally, areas of greater remoteness with more sparsely distributed populations may require an increased number of health professionals to account for the potentially larger distances of travel required. This means a single population to health practitioner ratio may not be appropriate for all levels of remoteness, which may also decrease the accuracy of the models created. However, to our knowledge more accurate minimum thresholds are not available, and the estimations supplied by the World Health Organisation are widely used.

Another limitation of this study was the inability to adjust for an LGA’s level of need. Areas of lower SES are likely to have increased burden of disease [25], and therefore may require more healthcare workers per head. Hence LGAs in lower IRSAD quintiles may be in deficit of healthcare workers due to increased demand, but in this analysis would not be classified as such if they are above the minimum threshold. This may bias the results towards the null, as was evident in findings from Fisher et al [23]. Further research into the association between SES and health workforce availability with adjustment for need may be valuable, and uncover associations that this paper was unable to.

It must also be noted that this analysis examines healthcare worker availability, and does not account for quality, acceptability, or accessibility of care. The number of FTE healthcare staff available is only a precondition to adequate care [32], with aspects of quality, acceptability and accessibility being equally important to the delivery of essential health care services [22,32].

## 6. Conclusion

Our findings suggest that local area-level SES is a determinant of workforce deficit among nurses and midwives, with the odds of being in deficit of nurses and midwives decreasing with decreasing SES. Additionally, we found that remote and very remote areas have decreased odds of being in deficit of nurses and midwives compared to inner regional areas. These findings suggest that current health workforce planning and policy relating to nurses and midwives may have been effective over the period of this study, as areas of higher need are less likely to be in deficit of these staff. Conversely, no association was found between local area-level SES and health workforce deficit among medical practitioners or allied health workers. However, it must be noted that this analysis does not account for increased need in areas of lower SES and in remote areas with geographical challenges.

## Supporting information

S1 File Supplementary materials. (DOCX)
