# Supplementary material for: Interlinkage between health workforce availability and socioeconomic status in rural and remote Australia
Source: PLoS One. 2025 Apr 11;20(4):e0321198. doi: 10.1371/journal.pone.0321198 (PMC11990773; doi:10.1371/journal.pone.0321198)
Supplement: S1 File — (DOCX) [file pone.0321198.s001.docx]

Table A: Results from the final Generalised Additive Model with Generalised Estimating Equations for each healthcare profession listed, with all covariates included and with comparison to each profession’s null model.

|  | Allied health professionals | | Nurses and midwives | | Medical practitioners | |
| --- | --- | --- | --- | --- | --- | --- |
|  | **Null** | **Final** | **Null** | **Final** | **Null** | **Final** |
| IRSAD^#^ | OR(SE)^##^ | OR(SE)^##^ | OR(SE)^##^ | OR(SE)^##^ | OR(SE)^##^ | OR(SE)^##^ |
| Quintile 2  Quintile 3  Quintile 4  Quintile 5 | -  -  -  - | 1.01(0.39)  0.96(0.42)  0.70(0.47)  0.65(0.56) | -  -  -  - | 1.34(0.16).  1.43(0.18).  1.65(0.20)*  2.54(0.29)** | -  -  -  - | 0.94(0.30)  1.12(0.34)  0.81(0.35)  0.93(0.41) |
| Area remoteness |  |  |  |  |  |  |
| Outer regional  Remote  Very remote |  | 1.39(0.42)  0.65(0.47)  0.30(0.46)** |  | 0.64(0.32)  0.12(0.61)**  0.28(0.41)** |  | 3.04(0.47)  1.56(0.50)  1.18(0.44) |
| Year | **-** | - | - | 1.01(0.01) | - | - |
| Intercept  OR(SE) | 11.75*** (0.14) | 26.50(0.51) | 0.17 (0.14) | 0.15(0.25) | 9.12(0.15) | 8.74(0.35) |
|  | **edf^###^** | **edf^###^** | **edf^###^** | **edf^###^** | **edf^###^** | **edf^###^** |
| f(Year)^####^ | - | 4.33*** | - | - | - | 1.58** |
| f(Proportion aged over 85 / 1000)^####^ | - | 7.72*** | - | 3.91*** | - | 8.01*** |
| f(Proportion aged under 5 / 1000)^####^ | - | 8.19*** | - | 8.23*** | - | 8.12*** |
| Performance statistics | | | | | | |
| C statistic | 0.5 | 0.77 | 0.5 | 0.76 | 0.5 | 0.73 |
| QIC | 1824 | 1652 | 2740 | 2331 | 2140 | 2016 |

**Significance key:** (p<0.001 = ***) (p<0.01 = **) (p<0.05 = *) (p<0.1 = .) (p≥0.1 = no symbol)

# IRSAD = Index of relative socioeconomic advantage and disadvantage

## OR(SE) = Odds ratio(standard error)

### edf = estimated degrees of freedom

#### f(x) = functional form of x

Table B: Crude logistic regression between index of relative socioeconomic advantage and disadvantage (IRSAD) quintiles.

| Crude logistic regression – Allied Health Professionals | | | | | | | | | |  |
| --- | --- | --- | --- | --- | --- | --- | --- | --- | --- | --- |
| Variable | | **Odds ratio** | **Standard error** | | | **Z value** | | **P value** | |  |
| IRSAD Quintiles  Quintile 2  Quintile 3  Quintile 4  Quintile 5 | | 0.88  0.72  0.62  0.44 | 0.21  0.20  0.20  0.24 | | | -0.59  -1.62  -2.41  -3.51 | | 0.554  0.105  0.016  <0.001 | |  |
| Intercept | | 15.89 | 0.15 | | | 18.00 | | <0.001 | |  |
| Crude logistic regression – medical practitioners | | | | | | | | | | |
| Variable | **Odds ratio** | | | **Standard error** | **Z value** | | | | **Significance** | |
| IRSAD Quintiles  Quintile 2  Quintile 3  Quintile 4  Quintile 5 | 1.79  1.11  1.14  1.18 | | | 0.18  0.16  0.17  0.23 | 3.24  0.67  0.76  0.72 | | | | ** | |
| Intercept | 7.54 | | | 0.11 | 17.91 | | | | *** | |
| Crude logistic regression – nurses and midwives | | | | | | | | | | |
| Variable | **Odds ratio** | | | **Standard error** | | | **Z value** | | **Significance** | |
| IRSAD Quintiles  Quintile 2  Quintile 3  Quintile 4  Quintile 5 | 0.91  1.06  2.00  7.62 | | | 0.17  0.17  0.16  0.17 | | | -0.56  0.33  4.43  11.86 | | ***  *** | |
| Intercept | 0.11 | | | 0.12 | | | -18.19 | | *** | |

**Significance key:** (p<0.001 = ***) (p<0.01 = **) (p<0.05 = *) (p<0.1 = .) (p≥0.1 = no symbol)

Table C: Logistic regression between index of relative socioeconomic advantage and disadvantage (IRSAD) quintiles, adjusting for remoteness, age distributions and year.

| Adjusted logistic regression – allied health professionals | | | | | | | | | | | | |  |  |
| --- | --- | --- | --- | --- | --- | --- | --- | --- | --- | --- | --- | --- | --- | --- |
| Variable | **Odds ratio** | | | **Standard error** | | | **Z value** | | | **Significance** | | |  |  |
| IRSAD Quintiles  Quintile 2  Quintile 3  Quintile 4  Quintile 5 | 0.64  0.58  0.46  0.32 | | | 0.23  0.22  0.22  0.26 | | | -1.94  -2.42  -3.53  -4.37 | | | .  *  ***  *** | | |  |  |
| Remoteness Area  Outer regional  Remote  Very remote | 1.54  1.14  0.36 | | | 0.19  0.24  0.21 | | | 2.33  0.55  -4.85 | | | *  *** | | |  |  |
| Proportion aged under 5 years / 1000 | 1.0 | | | 0.00 | | | 0.69 | | |  | | |  |  |
| Proportion aged over 85 years /1000 | 1.0 | | | 0.01 | | | -0.13 | | |  | | |  |  |
| Year | 0.74 | | | 0.03 | | | -9.95 | | | *** | | |  |  |
| Intercept | 95.72 | | | 0.53 | | | 8.53 | | | *** | | |  |  |
| Adjusted logistic regression – medical practitioners | | | | | | | | | | | | | |  |
| Variable | | **Odds ratio** | | | | **Standard error** | | **Z value** | | | **Significance** | | |  |
| IRSAD Quintiles  Quintile 2  Quintile 3  Quintile 4  Quintile 5 | | 1.14  0.86  1.02  1.10 | | | | 0.20  0.18  0.18  0.25 | | 0.66  -0.82  0.12  0.38 | | |  | | |  |
| Area Remoteness  Outer regional  Remote  Very remote | | 2.06  1.37  0.94 | | | | 0.17  0.20  0.19 | | 4.29  1.58  -0.34 | | | *** | | |  |
| Number aged under 5 years / 1000 | | 0.99 | | | | 0.00 | | -3.96 | | | *** | | |  |
| Number aged over 85 years /1000 | | 1.01 | | | | 0.01 | | 1.72 | | | . | | |  |
| Years | | 0.93 | | | | 0.02 | | -3.07 | | | ** | | |  |
| Intercept | | 26.67 | | | | 0.46 | | 7.19 | | | *** | | |  |
| Adjusted logistic regression – nurses and midwives | | | | | | | | | | | | | | |
| Variable | | | **Odds ratio** | | **Standard error** | | | | **Z value** | | | **Significance** | | |
| IRSAD Quintiles  Quintile 2  Quintile 3  Quintile 4  Quintile 5 | | | 1.18  1.20  1.33  5.60 | | 0.19  0.19  0.18  0.20 | | | | 0.84  0.95  1.58  8.52 | | | *** | | |
| Remoteness Area  Outer regional  Remote  Very remote | | | 0.79  0.08  0.05 | | 0.14  0.28  0.26 | | | | -1.69  -9.02  -11.83 | | | .  ***  *** | | |
| Number aged under 5 years / 1000 | | | 0.98 | | 0.00 | | | | -7.73 | | | *** | | |
| Number aged over 85 years / 1000 | | | 0.85 | | 0.01 | | | | -16.24 | | | *** | | |
| Years | | | 1.00 | | 0.02 | | | | -0.16 | | |  | | |
| Intercept | | | 42.90 | | 0.49 | | | | 7.66 | | | *** | | |

**Significance key:** (p<0.001 = ***) (p<0.01 = **) (p<0.05 = *) (p<0.1 = .) (p≥0.1 = no symbol)
